# Sex Differences in Hepatic VLDLR Response to Protein Restriction and Its Relationship with Hepatic Lipid Accumulation

**DOI:** 10.3390/nu18193153

**Published:** 2026-09-24

**Authors:** Jiuxiang Cao, Hiromu Morimoto, Asako Takenaka

**Affiliations:** Department of Agricultural Chemistry, School of Agriculture, Meiji University, Kawasaki 214-8571, Kanagawa, Japan

**Keywords:** VLDLR, protein restriction, sex differences, hepatic lipid accumulation, mice

## Abstract

**Background/Objectives**: The very low-density lipoprotein receptor (VLDLR) mediates VLDL uptake in peripheral tissues. This study investigated sex differences in hepatic VLDLR response to protein restriction and its relationship with hepatic lipid accumulation. **Methods**: Male and female wild-type (WT) and *Vldlr* knockout (KO) mice were fed control (20% protein) or low-protein (2% protein) diets for 20 days. Hepatic VLDLR expression and triglyceride levels were determined, followed by RNA sequencing and Gene Ontology (GO) analysis. **Results**: Hepatic *Vldlr* mRNA and protein levels were higher in females than in males. Protein restriction induced 9-fold and 2.5-fold increases in *Vldlr* mRNA and VLDLR protein, respectively, in males, whereas females showed only a 1.6-fold increase in VLDLR protein without significant *Vldlr* mRNA induction. The ATF4 target genes *Asns* and *Fgf21* responded similarly to protein restriction in both sexes. Although females had higher hepatic triglyceride levels than males, this difference persisted in *Vldlr* KO mice. RNA sequencing revealed sex-dependent effects of *Vldlr* knockout on hepatic gene expression, with inflammation- and immune-related terms predominantly identified in females. **Conclusions**: Sex differences in hepatic VLDLR expression do not account for sex differences in hepatic lipid accumulation. The sex-dependent VLDLR response to protein restriction may involve mechanisms other than differential ATF4 activation. *Vldlr* deficiency was associated with sex-dependent hepatic gene expression changes, particularly those involved in inflammation- and immune-related functions in females. These findings suggest that hepatic VLDLR may have functions beyond its established role in lipoprotein uptake and lipid metabolism and may be relevant to sex-dependent hepatic responses to low-protein nutrition.

## 1. Introduction

The very low-density lipoprotein receptor (VLDLR) is a member of the low-density lipoprotein receptor (LDLR) family and plays an important role in lipid transport through the uptake of triglyceride (TG)-rich lipoproteins [1]. VLDLR and LDLR share a five-domain structure but differ in their ligand specificity: LDLR primarily recognizes apolipoprotein (Apo) B-100, whereas VLDLR recognizes ApoE-containing chylomicrons, very low-density lipoprotein (VLDL), and intermediate-density lipoprotein [1,2]. Two alternatively spliced forms of *Vldlr* mRNA, type 1 and type 2, have been identified [3,4]. VLDLR is highly expressed in peripheral tissues, including the heart, skeletal muscle, and adipose tissue [5]. Increased VLDLR expression promotes lipid accumulation in adipose tissue and the heart [6,7], whereas *Vldlr*-deficient mice exhibit elevated plasma TG levels and reduced adipose tissue mass and body weight [2,8,9]. These findings indicate that VLDLR contributes to the uptake of TG-rich lipoproteins by peripheral tissues through lipoprotein lipase-mediated TG hydrolysis and receptor-mediated endocytosis [2,10].

Compared with adipose tissue, heart, and skeletal muscle, VLDLR expression is relatively low in the liver [3,4]. Nevertheless, hepatic VLDLR has been implicated in hepatic lipid accumulation. Hepatic *Vldlr* expression increases in response to endoplasmic reticulum (ER) stress, and both *Vldlr*-deficient and ApoE-deficient mice are protected from ER stress-induced hepatic steatosis, suggesting that VLDLR- and ApoE-dependent lipid uptake contributes to hepatic fat accumulation under these conditions [11]. Changes in hepatic VLDLR expression have also been reported in models of alcoholic and nonalcoholic fatty liver disease, including those involving peroxisome proliferator-activated receptor γ/δ and fibroblast growth factor 21 [12,13]. These findings suggest that hepatic VLDLR may contribute to the regulation of hepatic lipid accumulation. Moreover, recent studies have reported that hepatic *Vldlr* mRNA levels are negatively associated with coronary heart disease risk and that hepatic *Vldlr* upregulation promotes the clearance of atherogenic lipoproteins from the circulation and protects against atherosclerosis [14,15]. Thus, hepatic VLDLR may have metabolic effects that extend beyond its potential role in hepatic lipid accumulation.

In addition to its role in lipoprotein metabolism, VLDLR has been implicated in inflammatory processes. Increased VLDLR expression in macrophages promotes inflammation and impairs glucose tolerance in obese mice [16], whereas *Vldlr* deficiency attenuates high-fat diet-induced adipose tissue inflammation [17]. VLDLR-mediated inflammatory responses have also been linked to the interaction of VLDLR with fibrin, which facilitates leukocyte transmigration across the endothelial layer into tissues [18,19]. These findings suggest that VLDLR may participate in inflammatory and immune-related processes in addition to lipid transport. However, the functions of VLDLR in the liver, particularly in relation to hepatic lipid metabolism and inflammatory or immune-related processes, remain incompletely understood.

In our previous study, we found that protein deficiency markedly increases hepatic VLDLR mRNA levels in mice [20]. Protein deficiency is a characteristic feature of protein malnutrition and can cause hepatic lipid accumulation as well as loss of muscle mass. We therefore investigated whether the increase in hepatic VLDLR contributes to protein deficiency-induced hepatic lipid accumulation using *Vldlr* knockout (KO) mice. However, hepatic lipid accumulation increased to a similar extent in *Vldlr* KO and wild-type (WT) mice under protein-deficient conditions, indicating that VLDLR is not required for protein deficiency-induced hepatic lipid accumulation [20]. However, our previous study was conducted using male mice, and whether the regulation and potential functions of hepatic VLDLR differ between sexes remains unclear. Therefore, in the present study, we investigated sex differences in the response of hepatic VLDLR to protein restriction and examined whether VLDLR contributes to sex differences in hepatic lipid accumulation. Our previous study showed that a 3% protein diet increased plasma AST levels in *Vldlr* KO mice, although the effects on ALT levels were less clear [20]. Therefore, in the present study, we used a more severe protein-restricted condition (2% casein) to further clarify the effects of protein restriction and *Vldlr* deficiency on hepatic lipid accumulation and plasma markers of liver injury. We further analyzed hepatic gene expression using RNA sequencing to explore sex-dependent effects of VLDLR deficiency on hepatic gene expression.

## 2. Materials and Methods

### 2.1. Animals

Mice homozygous for the *Vldlr* targeted null mutation (*Vldlr*−/−) were purchased from The Jackson Laboratory (strain B6;127S7-*Vldlr*<tm1Her>/J, Stock No. 002529. Bar Harbor, ME, USA). These mice were crossed with C57BL/6J wild-type (WT) mice (Japan SLC, Inc., Shizuoka, Japan) to generate heterozygous (*Vldlr*+/−) mice. Heterozygous mice were subsequently crossed to obtain *Vldlr*−/− (KO) and WT mice for the experiments. Genotyping was performed after weaning as previously described, using the primers listed in Table 1. Mice were maintained at 22–24 °C under a 12 h light/dark cycle (06:00–18:00) with free access to tap water throughout the experimental period. Before the dietary intervention, mice were provided ad libitum access to a commercial pellet diet (MF; Oriental Yeast Co., Ltd., Tokyo, Japan).

### 2.2. Animal Experiments

Four-week-old male and female KO (*n* = 24) and WT (*n* = 24) mice were housed individually in stainless-steel cages with wire-mesh floors and without bedding material. Wire-mesh flooring was used to enable accurate measurement of food intake by preventing spilled diet from becoming mixed with bedding material. Animals were monitored regularly throughout the experimental period for general health and signs of distress. They were fed a control diet containing 20% casein as the protein source (20C; Table 2) for 3 days to acclimate them to powdered diets. Male and female KO and WT mice were then divided into two groups and fed either the 20C diet or a low-protein diet containing 2% casein (2C; Table 2) for 20 days. Six mice were assigned to each experimental group. Because food intake tended to decrease in KO mice fed the low-protein diet, pair-feeding was initiated on day 11 for both males and females. Food intake was matched to that of the KO-2C group, which exhibited the lowest food intake. On day 21, mice were anesthetized by inhalation of isoflurane (MSD Animal Health, Tokyo, Japan) using a small-animal anesthesia machine (MK-A100W; Muromachi Kikai Co., Ltd., Tokyo, Japan) under ad libitum feeding conditions, without fasting. Heparinized blood was collected by cardiac puncture, and plasma was obtained. The liver, heart, epididymal white adipose tissue (eWAT), and gastrocnemius muscle were excised, immediately frozen in liquid nitrogen, and stored at −80 °C until analysis. All animal experiments were approved by the Meiji University Institutional Animal Care and Use Committee (Approval No. IACUC 15-0007) and conducted in accordance with the guidelines of Meiji University. The study was conducted in accordance with the ARRIVE guidelines.

### 2.3. RNA Extraction and Real-Time PCR

Total RNA was extracted from tissues using TriPure Isolation Reagent (Roche Applied Science, Mannheim, Germany). cDNA synthesis and real-time PCR were performed using the PrimeScript RT Reagent Kit with gDNA Eraser (Perfect Real Time) and THUNDERBIRD SYBR qPCR Mix (Toyobo, Osaka, Japan), respectively, according to the manufacturers’ instructions [21]. *Actb* (β-actin) was used as the internal control. The amplification of a single PCR product for each primer set was confirmed by melting curve analysis. Gene expression levels were normalized to the mean value of the control group and expressed as relative mRNA levels. Primer sequences are listed in Table 1.

### 2.4. Protein Extraction and Western Blotting

Proteins were extracted from tissues as previously described, except that 250 μL of homogenization buffer was used for 70 mg eWAT samples [22]. Protein samples were immediately frozen in liquid nitrogen and stored at −80 °C until analysis. Sodium dodecyl sulfate-polyacrylamide gel electrophoresis and Western blotting were performed as previously described. The primary antibodies used were anti-mouse VLDLR antibody (R&D Systems, Minneapolis, MN, USA. AF2258; 1:10,000), anti-β-actin antibody (Abcam, Cambridge, UK. ab6276; 1:500), and anti-α-tubulin antibody (Abcam, Cambridge, UK. ab4074; 1:3000). The secondary antibodies were donkey anti-goat IgG-HRP (Santa Cruz, Dallas, Texas, USA. sc2020; 1:10,000), goat anti-mouse IgG-HRP (Abcam, Cambridge, UK. ab205719; 1:5000), and goat anti-rabbit IgG-HRP (Abcam, Cambridge, UK. ab6721; 1:10,000). Chemiluminescence was detected using Immobilon Western Chemiluminescent HRP Substrate (Merck, Darmstadt, Germany) and an ImageQuant LAS 4000 mini imaging system (GE Healthcare Life Sciences, Chicago, Illinois, USA). After VLDLR detection, membranes were stripped using Western BLoT Stripping Buffer (Takara, Kusatsu, Japan) and reprobed for β-actin or α-tubulin. Band intensities were quantified using ImageJ 1.54g software (National Institutes of Health). VLDLR levels were normalized to the mean value of the WT-20C group and expressed as relative protein levels. Densitometric analysis was performed using unmodified images. For samples analyzed on different membranes, quantitative values were standardized using the same control sample loaded on all gels.

### 2.5. Reverse Transcription PCR

The two alternatively spliced forms of *Vldlr* mRNA (types 1 and 2), generated by alternative splicing of exon 16, were measured by RT-PCR using Blend Taq (Toyobo, Osaka, Japan) and primers flanking exon 16, according to the method described by Sakai et al. (primer sequences are listed in Table 1). Total RNA was extracted from the liver, and cDNA was synthesized as described above. PCR amplification was performed for 30 cycles under conditions that did not reach the amplification plateau. The 300-bp and 200-bp products were designated as type 1 and type 2, respectively. Band intensities were quantified using ImageJ software (National Institutes of Health).

### 2.6. Analysis of Plasma and Liver Lipids

Total lipids were extracted from plasma and tissues using the method of Bligh and Dyer [23] and reconstituted in isopropanol. Triacylglycerol, total cholesterol, and phospholipid concentrations in plasma and total tissue lipid extracts were measured using the Triglyceride E-Test, Cholesterol E-Test, and Phospholipid C-Test, respectively (Wako Pure Chemical Industries, Osaka, Japan).

### 2.7. Markers of Liver Injury

Plasma aspartate aminotransferase (AST) and alanine aminotransferase (ALT) levels were measured by Oriental Yeast Co., Ltd. using the consensus method of the Japan Society of Clinical Chemistry (JSCC).

### 2.8. RNA Sequencing, Principal Component Analysis, and Gene Ontology Analysis

Total RNA was extracted from frozen liver samples and quantified by spectrophotometry. For each experimental group, RNA samples from six mice were pooled to generate one composite RNA sample, resulting in eight pooled RNA samples representing the eight experimental groups. RNA library preparation, RNA sequencing, and bioinformatic analyses were performed by Novogene Co., Ltd. (Beijing, China). RNA concentration and purity were assessed using a NanoDrop spectrophotometer (Thermo Fisher Scientific, Waltham, MA, USA), and RNA integrity was evaluated using an Agilent 2100 Bioanalyzer (Agilent Technologies, Santa Clara, CA, USA). mRNA was enriched from total RNA through poly(A) selection. cDNA libraries were constructed using cDNA synthesis, adapter ligation, and PCR amplification according to the manufacturer’s standard protocols. Library quality and concentration were assessed using a Qubit 2.0 fluorometer (Thermo Fisher Scientific, Waltham, MA, USA) and an Agilent 2100 Bioanalyzer (Santa Clara, CA, USA). Libraries were sequenced on an Illumina NovaSeq 6000 platform (Illumina, San Diego, CA, USA) using paired-end 150-bp reads. Raw FASTQ files were subjected to quality control using the standard Novogene pipeline to remove adapter sequences and low-quality reads. PCA was performed using normalized RNA-seq gene expression data from the eight pooled samples. The first two principal components (PC1 and PC2) were plotted to visualize the overall similarity and separation of gene expression profiles among the eight experimental groups. Differentially expressed genes (DEGs) were identified by comparing gene expression levels between experimental groups using the standard differential expression analysis pipeline provided by Novogene. The identified DEGs were functionally annotated and subjected to Gene Ontology (GO) enrichment analysis using the GO database.

### 2.9. Statistical Analysis

Two-way analysis of variance (ANOVA) was performed to evaluate the effects of *Vldlr* genotype and dietary protein level and their interaction. For analyses in which sex was included as a factor, two-way ANOVA was performed to evaluate the effects of sex and Vldlr genotype and their interaction under the control diet, or sex and dietary protein level and their interaction. Outliers were identified using the Smirnov-Grubbs test implemented in the BellCurve add-in for Excel, and data points identified as outliers were excluded from subsequent statistical analyses. When a significant interaction was detected (*p* < 0.05), multiple comparisons (Tukey–Kramer or Steel Dwass) were performed to evaluate differences between individual groups. Statistical significance was set at *p* < 0.05. All statistical analyses were performed using Excel 2016 with the BellCurve add-in.

## 3. Results

### 3.1. Animal Characteristics

The initial body weights of both male and female *Vldlr* KO mice were lower than those of WT mice and remained lower throughout the experimental period (Figure 1A,B). Protein restriction reduced body weight gain in both sexes, with a particularly marked reduction in *Vldlr* KO males (Figure 1A,B). During the pair-fed period, food intake did not differ significantly according to *Vldlr* genotype or dietary protein level (Figure 1C,D). Liver and gastrocnemius muscle weights were significantly reduced by protein restriction in both sexes, whereas BAT weight was significantly lower in *Vldlr* KO in males (Table 3).

### 3.2. Vldlr mRNA and VLDLR Protein Levels in the Liver, Muscle, and Heart

*Vldlr* mRNA and VLDLR protein were below detectable levels in the liver, muscle, and heart of *Vldlr* KO mice (Figure 2A–F). In WT males, protein restriction significantly increased hepatic *Vldlr* mRNA and VLDLR protein levels (Figure 2A,D). In females, protein restriction significantly increased hepatic VLDLR protein levels but did not significantly increase *Vldlr* mRNA levels (Figure 2A,D). Thus, the increases in hepatic *Vldlr* mRNA and VLDLR protein induced by protein restriction were less pronounced in females than in males (Figure 2A,D). Under the control (20C) diet, hepatic *Vldlr* mRNA and VLDLR protein levels were significantly higher in females than in males (Figure 2A,D). In contrast, *Vldlr* mRNA and VLDLR protein levels in muscle and heart were not significantly affected by protein restriction in either sex, and no significant sex differences were observed (Figure 2B,C,E,F).

RT-PCR analysis showed that both type 1 and type 2 *Vldlr* mRNA levels were higher in females than in males and were significantly increased by protein restriction (Figure 3).

The mRNA levels of *Asns* and *Fgf21*, which were measured as target genes of activating transcription factor 4 (ATF4), a transcription factor involved in the regulation of hepatic *Vldlr* expression, were increased by protein restriction in both sexes, regardless of *Vldlr* genotype (Figure 4).

### 3.3. Liver and Plasma Lipids

Hepatic TG and total cholesterol (T-Cho) levels were increased by protein restriction in both sexes, and hepatic TG levels were also increased in *Vldlr* KO mice (Figure 5A,B). In males, the increases in hepatic TG and T-Cho levels induced by protein restriction were greater in *Vldlr* KO mice than in WT mice (Figure 5A,B). Regarding plasma lipids, TG concentrations decreased, whereas T-Cho concentrations increased, in response to protein restriction in both sexes (Figure 5D,E). Hepatic phospholipid (PL) levels were slightly increased by *Vldlr* deficiency in protein-restricted males, whereas plasma PL levels were increased by protein restriction in females (Figure 5C,F). Sex differences in plasma and hepatic lipid levels were observed in mice fed the control diet (Figure 5A–F).

To examine the factors contributing to the changes in hepatic TG levels, we measured the expression of genes encoding enzymes involved in fatty acid synthesis and β-oxidation. Protein restriction increased the expression of genes encoding enzymes involved in fatty acid synthesis in both sexes (Figure 6A–C), whereas the expression of genes encoding enzymes involved in fatty acid oxidation was not significantly affected (Figure 6D,E).

### 3.4. Plasma AST and ALT Levels

Plasma AST and ALT concentrations, which are commonly used biochemical markers associated with hepatic injury, increased in response to protein restriction in male mice, regardless of *Vldlr* genotype, whereas these increases were not observed in females (Figure 7).

### 3.5. PCA and GO Enrichment Analysis of RNA-Sequencing Data

Principal component analysis (PCA) showed that PC1 and PC2 accounted for 48.4% and 18.7% of the total variance, respectively (Figure 8). Samples from the 2C groups were clearly separated from those from the 20C groups, primarily along PC1, irrespective of sex or *Vldlr* genotype, indicating that dietary protein restriction was a major source of variation in the overall hepatic gene expression profiles. Under the 20C condition, female samples were clearly separated from male samples. In addition, the effect of *Vldlr* deficiency appeared to be more pronounced in males than in females, as WT and KO males were clearly separated, whereas WT and KO females were closely positioned.

GO enrichment analysis was performed on genes differentially expressed between WT and *Vldlr* KO mice. The 30 most significant terms (adjusted *p* < 0.05) were selected for display; when fewer than 30 significant terms were identified, all significant terms were displayed (Figure 9). In males, 20 and 34 GO terms were enriched in the 20C and 2C groups, respectively (Figure 9A,B). Under the 20C condition, terms related to cell membrane transporters and channels were enriched (Figure 9A). Under the 2C condition, terms related to sterol and fatty acid metabolism were enriched in the biological process (BP) and molecular function (MF) categories (Figure 9B). In females, 49 and 177 GO terms were enriched in the 20C and 2C groups, respectively (Figure 9C,D). Terms related to inflammation and immune responses were enriched in the BP category under both dietary conditions (Figure 9C,D).

## 4. Discussion

### 4.1. Sex Differences in Hepatic VLDLR Response to Protein Restriction

We previously reported that protein restriction increases hepatic *Vldlr* mRNA and VLDLR protein levels in male mice [20]. These findings were confirmed in the present study. However, protein restriction did not significantly increase hepatic *Vldlr* mRNA levels in female mice, and the increase in VLDLR protein levels was less pronounced than that observed in males. Furthermore, hepatic *Vldlr* mRNA and VLDLR protein levels were significantly higher in females than in males under both dietary conditions. Sex differences in *Vldlr* mRNA levels were observed for both splice variants, type 1 and type 2. Although differences between these variants have been reported in tissue distribution [3,4], affinity for VLDL [3], and secretion into the circulation [24,25], the present results indicate that the observed sex difference was not restricted to a specific *Vldlr* splice variant.

Taken together, these findings demonstrate that hepatic *Vldlr* mRNA and VLDLR protein levels are higher in females than in males, whereas the induction of hepatic VLDLR by protein restriction is greater in males. A recent study reported that hepatic VLDLR expression is higher in females and that the relative increase in VLDLR expression following fenofibrate treatment is generally greater in males than in females [15]. This sex-dependent response to fenofibrate is consistent with the more pronounced induction of hepatic VLDLR by protein restriction observed in males in the present study. The increase in hepatic VLDLR expression upon protein restriction is predicted to depend on the transcription factor ATF4 [11]. The hepatic mRNA levels of *Asns* and *Fgf21*, target genes of ATF4, increased similarly in both sexes following protein restriction, suggesting that ATF4 activation occurred to a similar extent in males and females. Therefore, the sex difference in the induction of hepatic VLDLR expression by protein restriction is unlikely to be attributable to a sex difference in ATF4 activation.

### 4.2. Potential Mechanisms Underlying Sex Differences in Hepatic VLDLR Expression

Females had higher hepatic *Vldlr* mRNA and VLDLR protein levels than males under both dietary conditions. The mechanism underlying this sex difference remains unclear. One possible explanation is the sexually dimorphic pattern of growth hormone (GH) secretion [26]. GH signaling through signal transducer and activator of transcription 5 (STAT5) and the transcription factor B-cell lymphoma 6 (BCL6) contributes to sex-dependent patterns of hepatic gene expression [27]. *Vldlr* expression has been suggested to be regulated by STAT5 [28]. As no sex differences were observed in the expression of other ATF4 target genes, the observed sex difference in hepatic *Vldlr* mRNA abundance may involve an ATF4-independent mechanism, potentially involving STAT5-dependent regulation. Thus, sexually dimorphic GH–STAT5 signaling may contribute to the observed sex difference in hepatic *Vldlr* expression. However, this possibility was not directly examined in the present study and requires further investigation. The relatively high basal expression of *Vldlr* in females may also contribute to the smaller relative increase in response to protein restriction.

### 4.3. Hepatic VLDLR Does Not Account for the Sex Difference in Hepatic Lipid Accumulation

We previously reported that hepatic VLDLR abundance is lower than that in the heart, skeletal muscle, and adipose tissue [20]. Although hepatic VLDLR expression is relatively low, increased hepatic VLDLR has been reported to be associated with hepatic lipid accumulation in several experimental models [11,13,29]. For example, ER stress-induced hepatic steatosis is attenuated in *Vldlr* KO mice, suggesting that VLDLR-mediated lipid uptake contributes to hepatic fat accumulation under ER stress [11]. In contrast, our previous study showed that protein restriction-induced hepatic lipid accumulation was not prevented by *Vldlr* deficiency in male mice, indicating that increased VLDLR is not required for hepatic steatosis under protein restriction [20].

In the present study, protein restriction increased hepatic TG levels in both male and female *Vldlr* KO mice. Thus, the increase in hepatic lipid accumulation induced by protein restriction was not prevented by VLDLR deficiency in either sex. These findings further support the conclusion that the increase in hepatic VLDLR induced by protein restriction is not a primary determinant of hepatic lipid accumulation in this model. Other mechanisms may contribute to hepatic lipid accumulation, including reduced hepatic lipid secretion [30,31] and increased fatty acid synthesis [32,33]. Consistent with the latter possibility, protein restriction significantly increased the hepatic mRNA levels of *Acc-1*, *Acc-2*, and *Fas* in the present study (Figure 6), suggesting that enhanced de novo lipogenesis may contribute to hepatic TG accumulation under protein-restricted conditions. However, the similar hepatic TG levels between WT and *Vldlr* KO females, despite differences in the expression of these lipogenic genes, indicate that changes in de novo lipogenesis alone are unlikely to fully account for hepatic TG accumulation. Thus, other mechanisms may also contribute to the hepatic TG accumulation induced by protein restriction.

The higher hepatic TG levels observed in females than in males under the control diet initially raised the possibility that the higher hepatic VLDLR levels in females might contribute to this sex difference. However, the sex difference in hepatic TG levels persisted in *Vldlr* KO mice. Therefore, hepatic VLDLR expression does not appear to account for the sex difference in hepatic lipid accumulation. Although recent studies have suggested sex differences in the hepatic effects of FGF21 on lipid metabolism [34], the mechanisms underlying the sex difference in hepatic lipid accumulation remain to be determined. Another potential mechanism relevant to the sex difference in hepatic lipid metabolism is phosphatidylcholine metabolism mediated by phosphatidylethanolamine *N*-methyltransferase (PEMT). PEMT catalyzes the methylation of phosphatidylethanolamine to phosphatidylcholine and is involved in hepatic phospholipid metabolism and VLDL secretion [35,36]. Sex-dependent differences in hepatic PEMT expression and in the metabolism of PEMT-derived phosphatidylcholine have also been reported [37]. In the present RNA-seq dataset, *Pemt* mRNA expression appeared higher in females than in males under the control condition. Protein restriction was associated with an apparent increase in *Pemt* expression in males and a smaller increase in females, and a similar pattern was observed in Vldlr KO mice. Interestingly, the pattern of *Pemt* expression broadly paralleled the changes in hepatic TG levels rather than showing an inverse relationship. This finding does not support a simple model in which increased PEMT expression directly limits hepatic TG accumulation through enhanced VLDL secretion. Instead, the induction of *Pemt* may represent a compensatory response to altered hepatic lipid metabolism during protein restriction. However, this possibility remains speculative because PEMT protein abundance, enzymatic activity, and hepatic VLDL secretion were not assessed in the present study. Furthermore, because the RNA-seq analysis was performed using pooled samples without biological replication, these observations should be considered exploratory.

In addition, protein restriction increased plasma AST and ALT levels in males but not in females. These findings indicate a sex-dependent increase in biochemical markers associated with hepatic injury; however, because histological examination was not performed in the present study, the presence or extent of structural liver injury cannot be determined from these measurements alone.

### 4.4. Sex-Dependent Effects of VLDLR Deficiency on Hepatic Gene Expression

We further analyzed hepatic gene expression using RNA sequencing and performed PCA and GO enrichment analyses to examine the effects of VLDLR deficiency on hepatic gene expression. PCA showed that the effect of *Vldlr* deficiency on the overall hepatic gene expression profiles appeared to be relatively small in females but more pronounced in males. In contrast, the 2C groups were clearly separated from the 20C groups irrespective of sex and genotype, indicating that dietary protein restriction had a major influence on the overall hepatic gene expression profiles.

GO enrichment analysis of genes differentially expressed following *Vldlr* knockout revealed distinct functional patterns between sexes. In males, terms related to cell membrane transporters and channels were enriched under the control condition, whereas terms related to sterol and fatty acid metabolism were enriched under the protein-restricted condition. In females, inflammation- and immune-related terms were prominently enriched under both dietary conditions. Thus, although the overall effect of *Vldlr* deficiency on hepatic gene expression profiles appeared to be more pronounced in males based on PCA, *Vldlr* deficiency was associated with prominent changes in inflammation- and immune-related functional categories in females, indicating that the functional consequences of *Vldlr* deficiency differed between sexes.

VLDLR has been implicated in inflammatory processes, including leukocyte transendothelial migration through its interaction with fibrin [19]. In addition, the liver contains not only hepatocytes but also non-parenchymal cells, including sinusoidal endothelial cells and Kupffer cells, that participate in inflammatory and immune responses. Thus, the enrichment of inflammation- and immune-related GO terms in females suggests that *Vldlr* deficiency is associated with alterations in the expression of genes related to inflammatory and immune processes. However, these findings do not establish the presence of hepatic inflammation or immune activation because the present study did not directly measure inflammatory or immune markers. Moreover, the present RNA-seq analysis was performed using pooled samples, and therefore the GO analysis was based on group-level gene expression changes without biological replication. Accordingly, these findings should be interpreted as an indication of altered biological processes rather than direct evidence of hepatic inflammation or immune activation.

Taken together, the present findings suggest that the effects of VLDLR deficiency on hepatic gene expression differ between sexes and are not primarily characterized by changes in lipid metabolic pathways. In females, changes in genes associated with inflammatory and immune-related processes were particularly prominent, suggesting that hepatic VLDLR may have functions beyond its established role in lipoprotein uptake and lipid metabolism.

### 4.5. Limitations of the Study

A limitation of the present study is the use of pooled samples for RNA-seq analysis. Because liver RNA samples from individual mice within each experimental group were pooled to generate a single library per group, the RNA-seq data did not include true biological replication. Therefore, the differential expression, PCA, and GO enrichment analyses should be interpreted as exploratory analyses of group-level gene expression patterns rather than as statistically replicated evidence of differential gene expression or pathway enrichment. In particular, the inflammation- and immune-related GO terms observed in females should not be interpreted as direct evidence of hepatic inflammation or immune activation, because inflammatory and immune markers were not directly assessed.

Several aspects of the experimental design may also have influenced the observed phenotypes. Individual housing, which was necessary for accurate measurement of food intake and implementation of pair-feeding, may have introduced social isolation-related stress. In addition, because pair-feeding was initiated on day 11 to match the reduced food intake of the KO-2C group, the other groups also experienced a secondary reduction in energy intake during the latter half of the experimental period. Although this approach minimized differences in food intake among groups, its potential contribution to the observed phenotypes cannot be completely excluded. Furthermore, the mice were placed on the experimental diets at 4 weeks of age and analyzed at approximately 7 weeks of age. Thus, growth and sexual maturation may have influenced the observed sex-dependent differences. Although sex-dependent hepatic gene expression patterns, including sex-biased expression of several genes associated with GH-dependent hepatic regulation, were already evident at approximately 7 weeks of age, these findings do not establish that adult-like endocrine patterns had been fully established.

Finally, liver histology was not assessed. Although plasma AST and ALT are commonly used biochemical markers associated with hepatic injury, these measurements alone cannot establish the presence or extent of histological liver injury. Therefore, the increases in plasma AST and ALT observed in male mice under protein restriction should not be interpreted as direct evidence of structural hepatic injury. Further studies incorporating biological replication of RNA-seq samples, direct assessment of inflammatory and immune markers, histological examination, and experimental designs that independently control dietary protein and energy intake will be necessary to confirm and extend the present findings.

## 5. Conclusions

In conclusion, hepatic *Vldlr* mRNA and VLDLR protein levels differed between sexes, with higher levels in females than in males. The increase in hepatic VLDLR in response to protein restriction was pronounced in males but modest in females. Because *Vldlr* deficiency did not abolish the sex difference in hepatic TG levels, the higher hepatic VLDLR levels in females do not appear to account for the observed sex difference in hepatic TG levels. Furthermore, the sex difference in the induction of hepatic VLDLR by protein restriction was not accompanied by a sex difference in the expression of ATF4 target genes, suggesting that this differential response may involve an ATF4-independent mechanism. RNA sequencing revealed sex-dependent effects of VLDLR deficiency on hepatic gene expression, with inflammation- and immune-related functional terms predominantly represented among the genes altered in females under both control and protein-restricted conditions. Collectively, these findings suggest that hepatic VLDLR is not a major determinant of hepatic lipid accumulation in this model and may have functions beyond its established role in lipid metabolism (Figure 10).

## Figures and Tables

**Figure 1:**
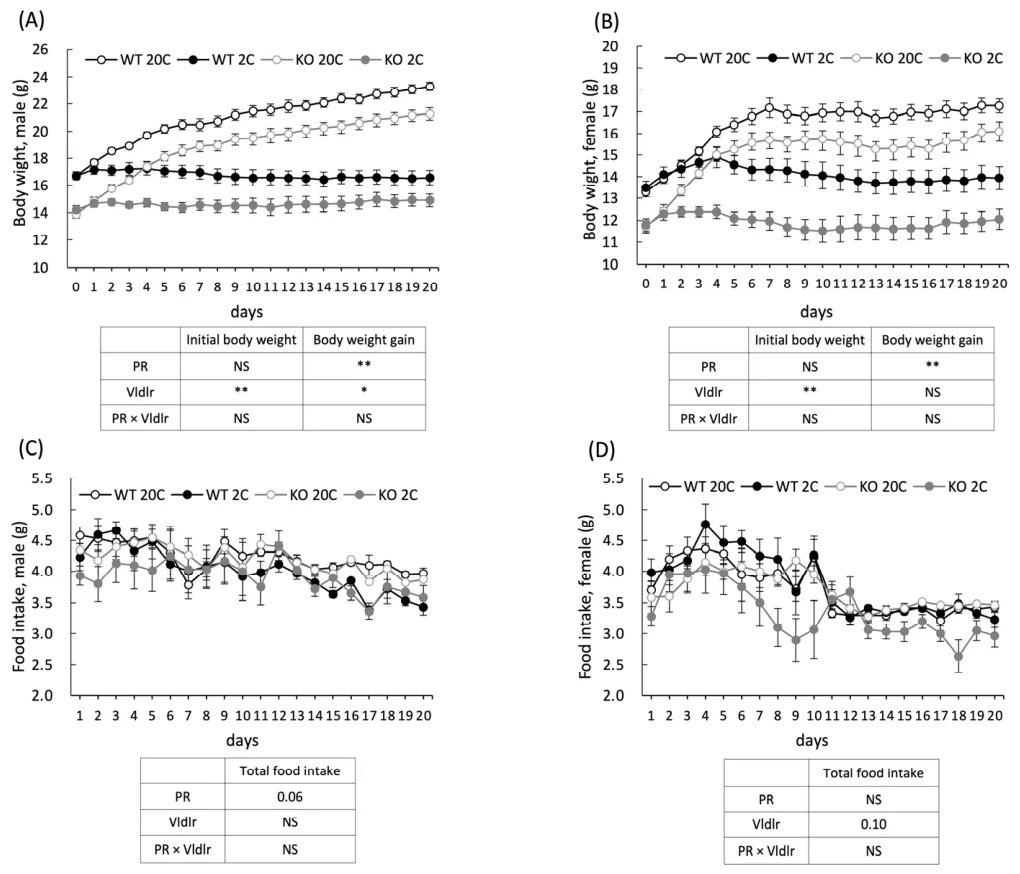
Effect of *Vldlr* knockout and protein restriction on mouse growth and food intake. (**A**) Body weight and (**C**) food intake in male mice; (**B**) body weight and (**D**) food intake in female mice. Wild-type (WT) and *Vldlr* knockout (KO) mice were fed a control (20C) or low-protein (2C) diet. Data are presented as mean ± SEM (*n* = 6 per group). Two-way ANOVA was performed to assess the effects of *Vldlr* deficiency, protein restriction (PR), and their interaction. ** *p* < 0.01, * *p* < 0.05; NS, not significant.

**Figure 2:**
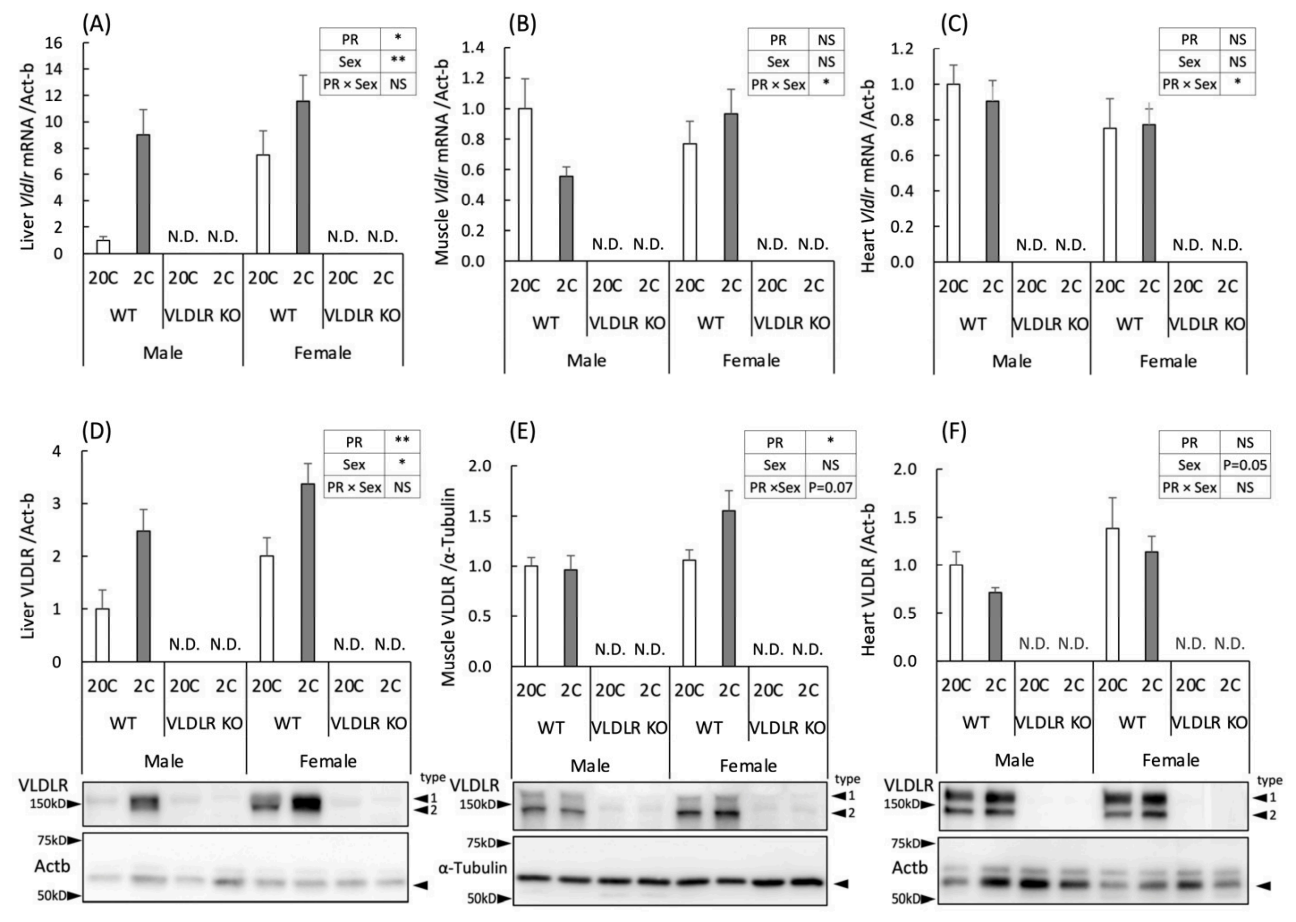
Effects of protein restriction and sex on *Vldlr* mRNA and VLDLR protein levels in the liver, gastrocnemius muscle, and heart. (**A**–**C**) Relative *Vldlr* mRNA levels in the liver (**A**), gastrocnemius muscle (**B**), and heart (C) of male and female wild-type (WT) mice fed a control (20C) or low-protein (2C) diet. (**D**–**F**) Relative VLDLR protein levels in the liver (**D**), gastrocnemius muscle (**E**), and heart (**F**), with representative Western blot images shown below the corresponding graphs. β-actin or α-tubulin was used as the loading control. Relative values were normalized to the mean value of the WT male 20C group, which was set to 1. Data are presented as mean ± SEM (*n* = 6 per group). ND, not detected. Two-way ANOVA was performed separately for each tissue to assess the effects of protein restriction (PR), sex, and their interaction (PR × sex). The results are shown in the tables adjacent to the graphs. Statistical significance was defined as ** *p* < 0.01, * *p* < 0.05; NS, not significant.

**Figure 3:**
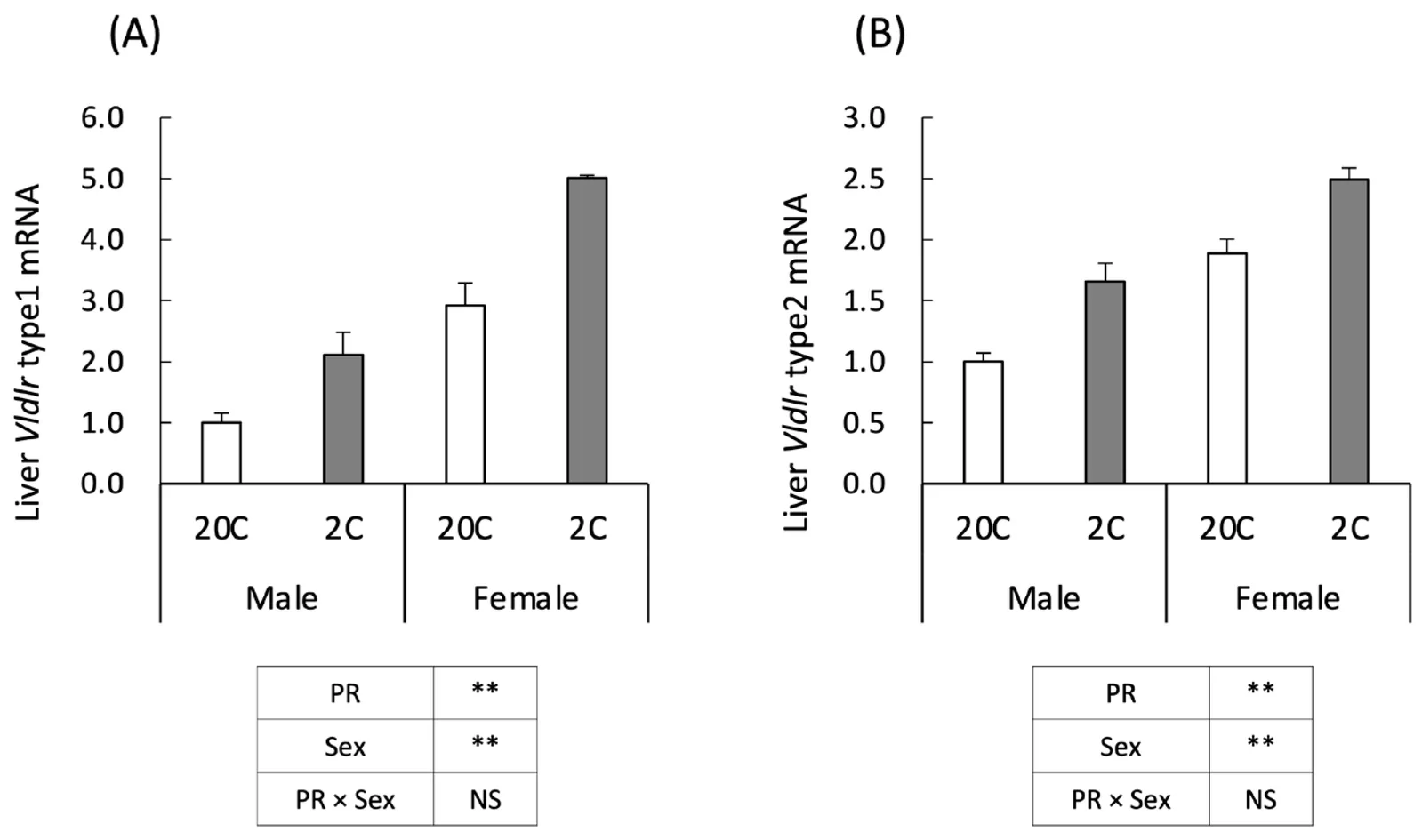
Effects of protein restriction and sex on type 1 and type 2 *Vldlr* mRNA expression. Relative type 1 (**A**) and type 2 (**B**) *Vldlr* mRNA levels in WT mice fed the control (20C) or protein-restricted (2C) diet. Relative values were normalized to the mean value of the male 20C group, which was set to 1. Values are presented as the mean ± SEM, with *n* = 6 per group. Results from a two-way ANOVA assessing the effects of protein restriction (PR), sex, and their interaction are depicted in the tables below the graphs; significance is denoted as ** *p* < 0.01, NS, not significant.

**Figure 4:**
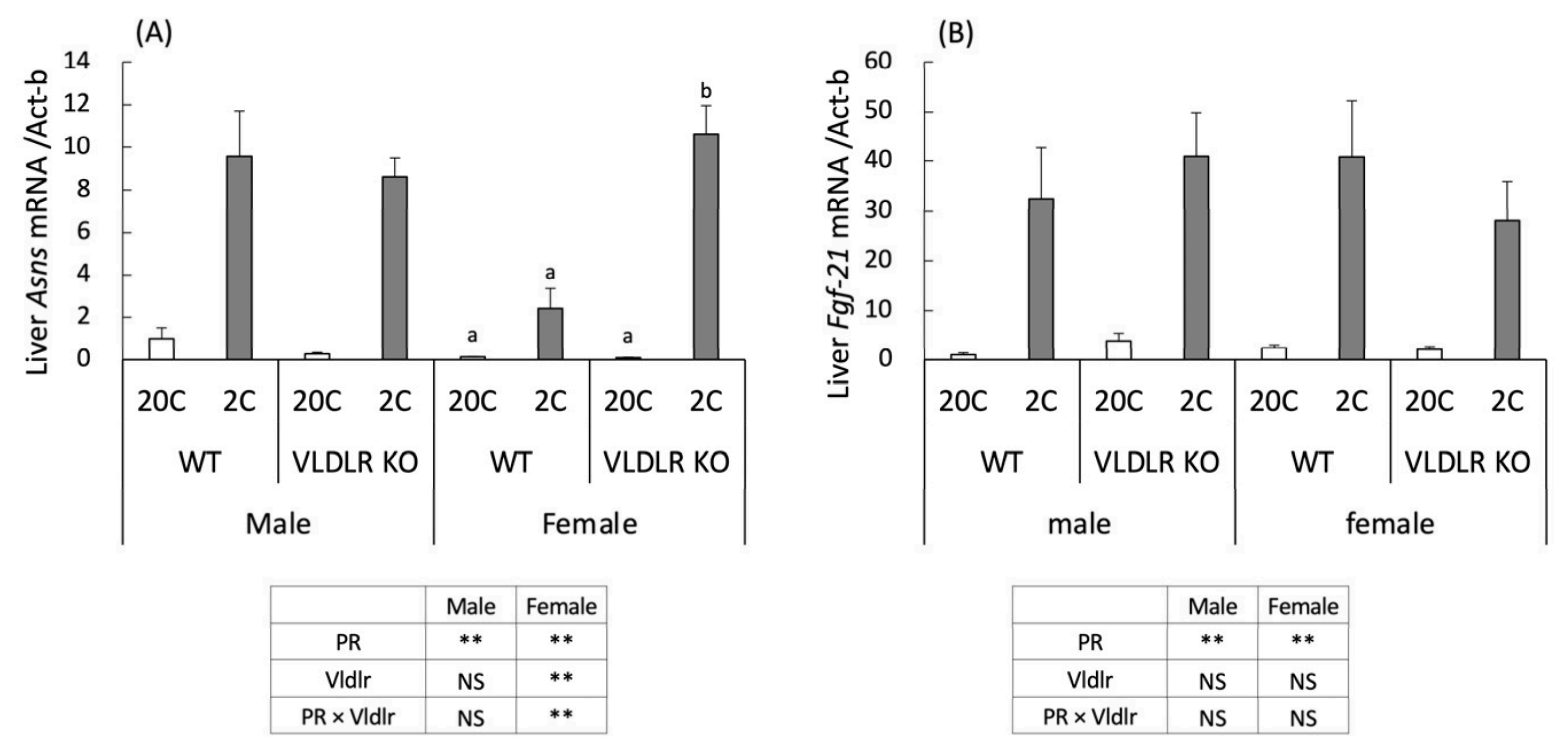
Effects of *Vldlr* knockout and protein restriction on *Asns* and *Fgf21* mRNA expression. Relative *Asns* (**A**) and *Fgf21* (**B**) mRNA levels in wild-type (WT) or *Vldlr*-deficient (KO) mice fed the control (20C) or protein-restricted (2C) diet. Relative values were normalized to the mean value of the WT male 20C group, which was set to 1. Values are presented as the mean ± SEM, with *n* = 6 per group. Results from a two-way ANOVA assessing the effects of *Vldlr* deficiency (Vldlr), protein restriction (PR), and their interaction are depicted in the tables below the graphs; significance is denoted as ** *p* < 0.01, NS, not significant. When a significant interaction was detected, multiple comparisons were performed, and significant differences are indicated by different letters.

**Figure 5:**
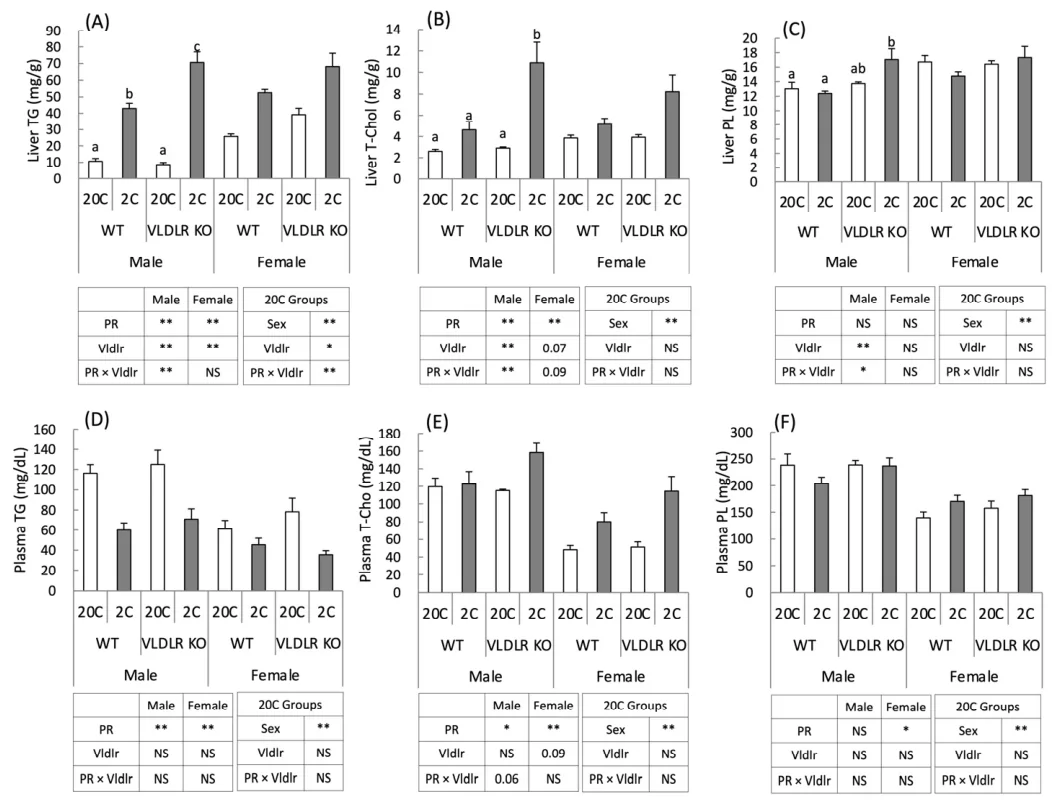
Effects of *Vldlr* knockout and protein restriction on hepatic and plasma lipid levels. (**A**,**D**) Triglyceride (TG), (**B**,**E**) total cholesterol (T-Cho), and (**C**,**F**) phospholipid (PL) levels in the liver and plasma, respectively, of WT and *Vldlr* KO mice fed a control (20C) or low-protein (2C) diet. Data are presented as mean ± SEM (*n* = 6 per group). Two-way ANOVA was performed separately in males and females to assess the effects of *Vldlr* deficiency (Vldlr), protein restriction (PR), and their interaction. For the 20C groups, the effects of sex, *Vldlr* deficiency (Vldlr), and their interaction were also analyzed. When a significant interaction was detected, multiple comparisons were performed, and significant differences are indicated by different letters. ** *p* < 0.01, * *p* < 0.05; NS, not significant.

**Figure 6:**
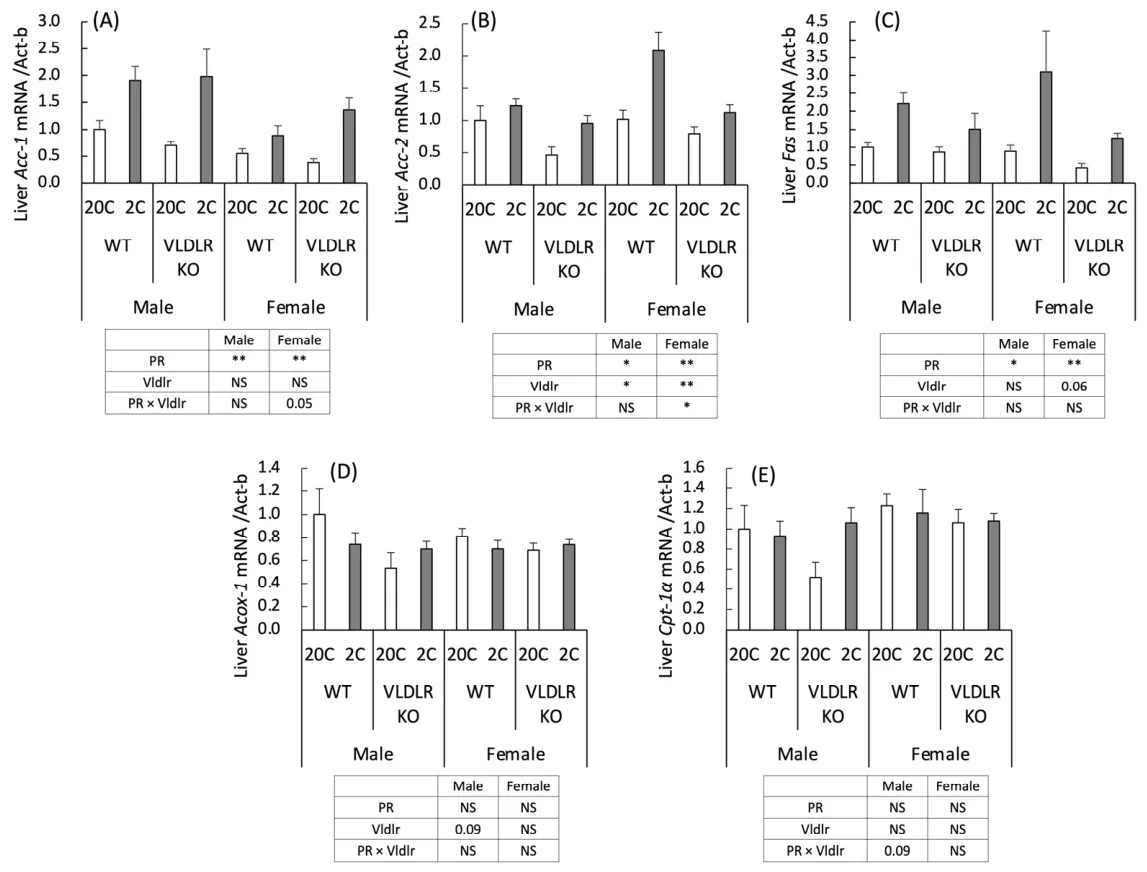
Effect of *Vldlr* knockout and protein restriction on fatty acid synthesis and β-oxidation in mice. Relative *Acc-1* (**A**), *Acc-2* (**B**), *Fas* (**C**), *Acox-1* (**D**), and *Cpt-1a* (**E**) mRNA levels in wild-type (WT) or *Vldlr*-deficient (KO) mice fed the control (20C) or protein-restricted (2C) diet. Relative values were normalized to the mean value of the WT male 20C group, which was set to 1. Values are presented as the mean ± SEM, with *n* = 6 per group. Results from a two-way ANOVA assessing the effects of *Vldlr* deficiency (Vldlr), protein restriction (PR), and their interaction are depicted in the tables below the graphs; significance is denoted as ** *p* < 0.01, * *p* < 0.05; NS, not significant.

**Figure 7:**
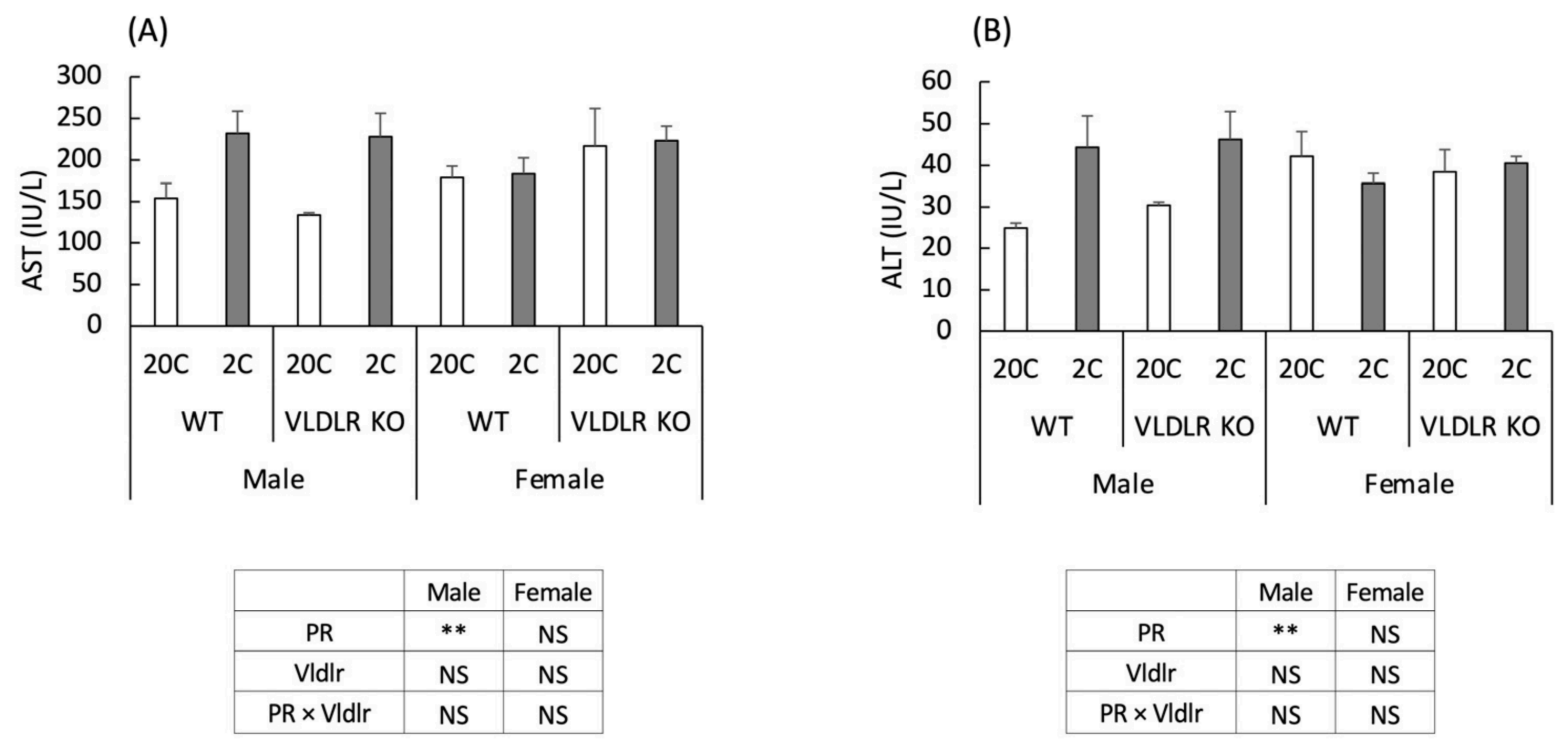
Effects of *Vldlr* knockout and protein restriction on plasma markers of hepatic injury. (**A**) Plasma aspartate aminotransferase (AST) and (**B**) alanine aminotransferase (ALT) levels in WT and *Vldlr* KO mice fed a control (20C) or low-protein (2C) diet. Data are presented as mean ± SEM (*n* = 6 per group). Two-way ANOVA was performed to assess the effects of *Vldlr* deficiency (Vldlr), protein restriction (PR), and their interaction. ** *p* < 0.01; NS, not significant.

**Figure 8:**
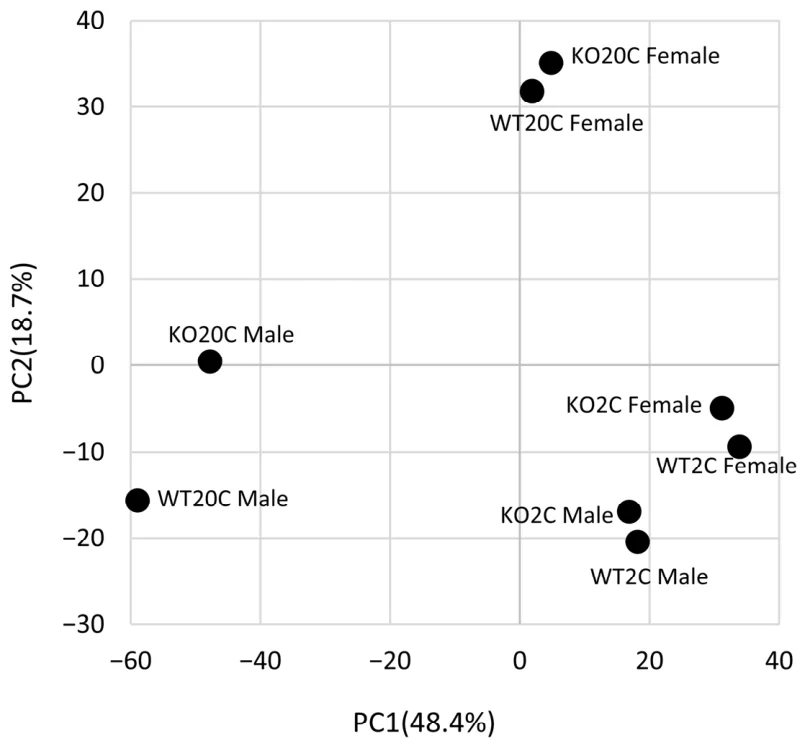
Principal component analysis (PCA) of hepatic RNA-seq gene expression profiles across the eight experimental groups. PCA was performed using normalized RNA-seq gene expression data from eight pooled RNA samples, with RNA from six mice pooled within each experimental group. Each point represents one pooled RNA sample. PC1 and PC2 accounted for 48.4% and 18.7% of the total variance, respectively. Samples are indicated by different colors according to experimental group.

**Figure 9:**
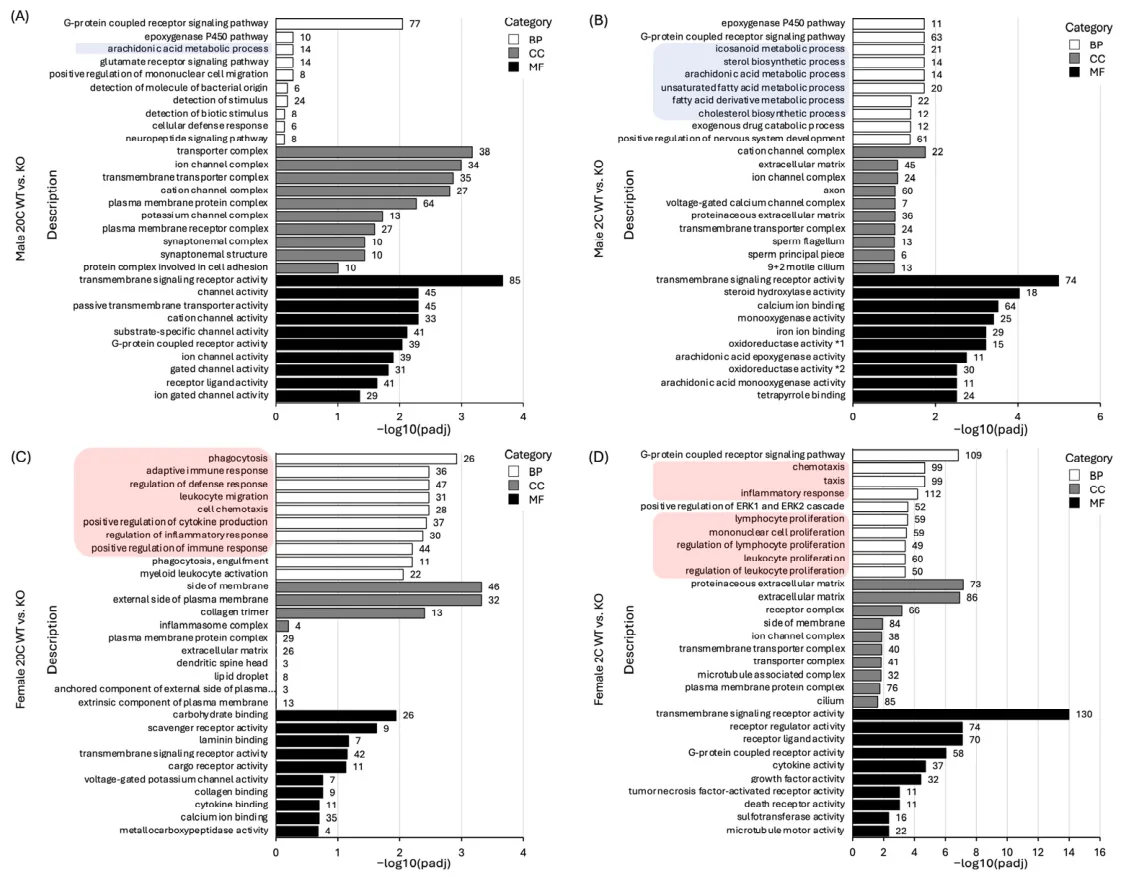
Gene Ontology (GO) enrichment analysis of genes differentially expressed between WT and *Vldlr* KO mice. GO enrichment analysis was performed for the following comparisons: (**A**) male 20C WT vs. KO, (**B**) male 2C WT vs. KO, (**C**) female 20C WT vs. KO, and (**D**) female 2C WT vs. KO. The 30 most significant GO terms were selected for display, or all significant terms were shown when fewer than 30 terms were identified. GO terms are categorized into biological process (BP), cellular component (CC), and molecular function (MF). The *x*-axis indicates −log10 (adjusted *p* value), and the number of differentially expressed genes is indicated by the data labels. In the BP category, terms related to sterol and fatty acid metabolism in (**B**) are highlighted in blue, whereas terms related to inflammation and immune responses in (**C**,**D**) are highlighted in red. *1: oxidoreductase activity on paired donors, with incorporation or reduction of molecular oxygen, reduced flavin, or flavoprotein as one donor, and incorporation of one oxygen atom. *2: oxidoreductase activity on paired donors with the incorporation or reduction of molecular oxygen.

**Figure 10:**
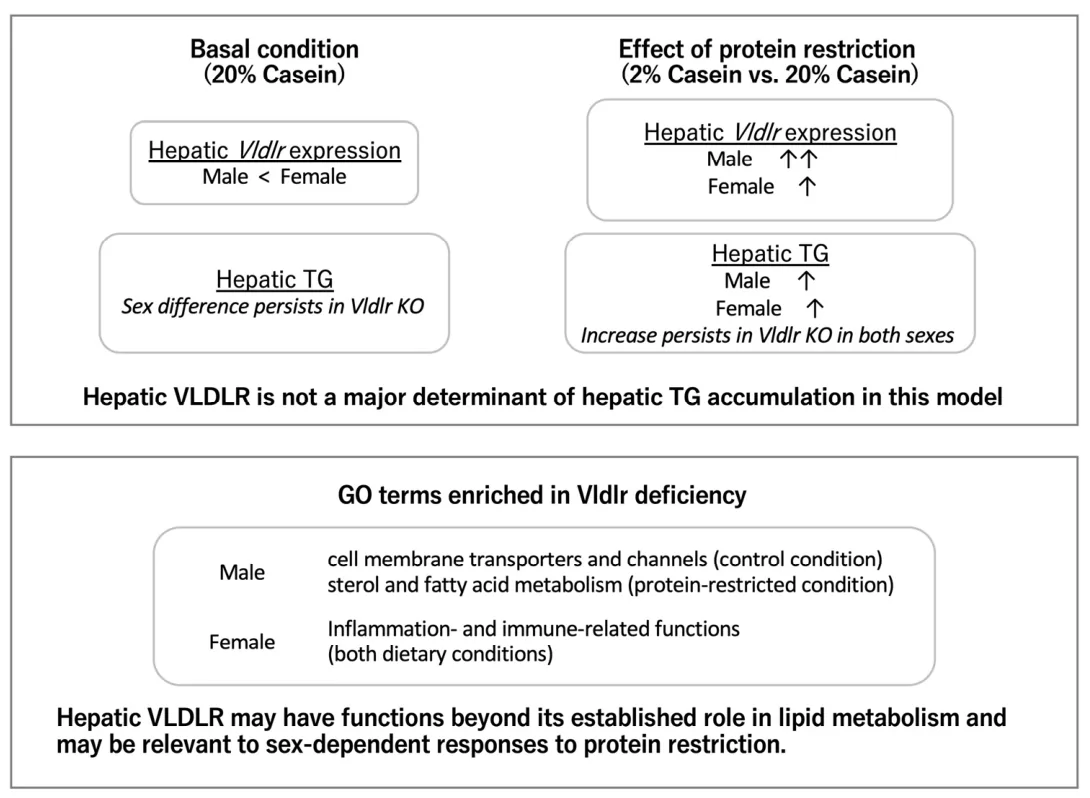
Proposed model summarizing the sex-dependent hepatic responses to protein restriction and *Vldlr* deficiency. Protein restriction increased hepatic VLDLR protein levels in both males and females, with a greater relative increase observed in males. Hepatic triglyceride (TG) levels were increased by protein restriction in both sexes, and this increase persisted in *Vldlr* knockout mice. The sex difference in hepatic TG levels also persisted in *Vldlr* knockout mice, indicating that the higher hepatic VLDLR levels in females do not account for the higher hepatic TG levels. RNA-seq analysis revealed sex-dependent effects of *Vldlr* deficiency on hepatic gene expression. In males, genes related to cell membrane transporters and channels under the control condition and sterol and fatty acid metabolism under the protein-restricted condition were predominantly represented, whereas inflammation- and immune-related functional terms were predominantly represented in females under both dietary conditions. Collectively, these findings suggest that hepatic VLDLR may have functions beyond its established role in lipid metabolism and may be relevant to sex-dependent hepatic responses to protein restriction.

**Table 1:** Primers for real-time PCR, RT-PCR and genotyping.

Gene Name	5′-Sequence-3′	Product Size (bp)
**Real-time PCR**		
Actb (β-actin)		
Forward	AAGTGTGACGTTGACATCCGTAA	90
Reverse	GCAATGCCTGGGTACATGGT	
Vldlr		
Forward	TGACGCAGACTGTTCAGACC	238
Reverse	GCCGTGGATACAGCTACCAT	
Acc-1		
Forward	TAAACCAGCACTCCCGATTC	80
Reverse	CCATCCTGTAAGCCAGAGAT	
Acc-2		
Forward	TGTGGCAGACGCGATGTT	80
Reverse	GCCGCCGTAAATCAATTCAA	
Fas		
Forward	TTCCAAGACGAAAATGATGC	80
Reverse	AATTGTGGGATCAGGAGAGC	
Acox-1		
Forward	GCCTTTGTTGTCCCTATCCGT	80
Reverse	CGATATCCCCAACAGTGATGC	
Cpt-1α		
Forward	AGACCGTGAGGAACTCAAACCTAT	80
Reverse	TGAAGAGTCGCTCCCACTGA	
**RT-PCR**		
Vldlr		
WT Forward	ATATCTCTGCCTGCCAGCACC	230 and 314
WT Reverse	TCCTCCACATCAAGTAGCCACC	
**Genotyping**		
Vldlr		
WT Forward	TGGTGATGAGAGGCTTGTATGTTGTC	400
WT Reverse	TTGACCTCATCGCTGCCGTCCTTG	
Mutant Forward	CGGCGAGGATCTCGTCGTGACCCA	200
Mutant Reverse	GCGATACCGTAAAGCACGAGGAAG	

**Table 2:** Composition of the experimental diet (g/100 g diet).

	20C	2C
casein	20	2
L-methionine	0.32	0.032
a-cornstarch	43.45	55.645
sucrose	21.73	27.823
cellulose	5	5
AIN93G vitamin mixture	1	1
AIN93 mineral mixture	3.5	3.5
corn oil	5	5
total	100	100

**Table 3:** Tissue weights of WT and *Vldlr* KO mice fed the control (20C) or low low-protein (2C) diet.

	WT		VLDLR KO		Two-Way ANOVA		
	20C	2C	20C	2C	PR	Vldlr	PR × Vldlr
**male**							
liver weight (g)	1.01 ± 0.06	0.64 ± 0.05	0.95 ± 0.04	0.64 ± 0.04	**	NS	NS
gastrocnemius weight (g)	0.23 ± 0.01	0.16 ± 0.01	0.22 ± 0.01	0.15 ± 0.01	**	NS	NS
soleus weight (mg)	9.90 ± 0.65	13.75 ± 7.51	8.78 ± 0.83	15.52 ± 7.19	NS	NS	NS
perirenal fat weight (mg)	27.33 ± 3.85	52.67 ± 32.54	28.00 ± 4.70	29.50 ± 5.68	NS	NS	NS
subcutaneous fat weight (g)	0.25 ± 0.02	0.24 ± 0.04	0.25 ± 0.02	0.18 ± 0.02	NS	NS	NS
BAT weight (mg)	89.83 ± 5.60	82.37 ± 9.25	76.75 ± 7.09	64.67 ± 6.07	NS	*	NS
**female**							
liver weight (g)	0.70 ± 0.02	0.53 ± 0.03	0.71 ± 0.04	0.45 ± 0.06	**	NS	NS
gastrocnemius weight (g)	0.15 ± 0.02	0.13 ± 0.00	0.15 ± 0.00	0.11 ± 0.01	*	NS	NS
soleus weight (mg)	6.82 ± 1.30	6.90 ± 0.73	11.35 ± 3.31	15.25 ± 10.57	NS	NS	NS
perirenal fat weight (mg)	47.12 ± 9.35	56.33 ± 11.38	60.83 ± 6.06	34.67 ± 7.09	NS	NS	NS
subcutaneous fat weight (g)	0.20 ± 0.03	0.23 ± 0.03	0.20 ± 0.02	0.17 ± 0.01	NS	NS	NS
BAT weight (mg)	65.58 ± 6.49	66.50 ± 7.38	62.17 ± 9.17	56.00 ± 5.87	NS	NS	NS

Two-way ANOVA was performed to assess the effects of *Vldlr* deficiency, protein restriction (PR), and their interaction. ** *p* < 0.01, * *p* < 0.05; NS, not significant.

## Data Availability

The datasets generated and/or analyzed during the current study are available in the Gene Expression Omnibus (GEO) repository (GSE259313).

## Abbreviations

The following abbreviations are used in this manuscript:

ANOVA	Analysis of variance
Apo	Apolipoprotein
ATF	Activating transcription factor
BAT	Brown adipose tissue
BCL6	B cell lymphoma 6
BP	Biological process
CC	Cell component
ER	Endoplasmic reticulum
FGF	Fibroblast growth factor
GEO	Gene Expression Omnibus
GH	Growth hormone
GO	Gene ontology
JSPS	Japan Society for the Promotion of Science
KO	Knockout
LDLR	Low-density lipoprotein receptor
MF	Molecular function
RT-PCR	Reverse transcription PCR
STAT5	Signal transducer and activator of transcription 5
TG	Triglycerides
VLDL	Very low-density lipoprotein
VLDLR	Very low-density lipoprotein receptor
WT	Wild-type
